# FAIR-Birth: Development and Feasibility Testing of an AI-Supported Advance Birth Planning Application for Midwifery-Led Antenatal Care—A Mixed-Methods Study Protocol

**DOI:** 10.3390/healthcare14121607

**Published:** 2026-06-08

**Authors:** Michaela Schunk, Christoph Hübener, Sebastian Robert, Sebastian P. Bayerl, Karolina Luegmair

**Affiliations:** 1 Fakultät für Angewandte Gesundheits- und Sozialwissenschaften, Technische Hochschule Rosenheim, 83024 Rosenheim, Germany; michaela.schunk@th-rosenheim.de (M.S.); sebastian.robert@th-rosenheim.de (S.R.); 2 Fakultät für Gesundheit und Pflege, Katholische Stiftungshochschule München, 81667 München, Germany; christoph.huebener@ksh-m.de; 3 Klinik und Poliklinik für Frauenheilkunde und Geburtshilfe, LMU Klinikum, LMU München, 81377 München, Germany; 4 Fakultät für Informatik, Technische Hochschule Rosenheim, 83024 Rosenheim, Germany; sebastian.bayerl@th-rosenheim.de

**Keywords:** advance birth planning, midwifery-led care, shared decision-making, artificial intelligence, large language models, health equity, feasibility study, multilingualism, antenatal care

## Abstract

**Background/Objectives**: Clinical childbirth in high-income countries is increasingly shaped by standardised routines that do not always accommodate individual preferences. In Germany, approximately one in eight pregnant persons experiences clinically significant childbirth-related post-traumatic stress disorder symptoms, with pregnant persons facing language or health-literacy barriers being at particular risk of inadequate preference integration. **Methods**: This paper presents the conceptual foundation and proposed study design for FAIR-Birth, an interdisciplinary initiative to develop and feasibility-test a mobile application supporting Advance Birth Planning (ABP) embedded within midwifery-led antenatal care. The intervention combines four elements: transfer of the Advance Care Planning concept to antenatal care, a domain-restricted Large Language Model (LLM) supporting multilingual preference articulation, integration of the resulting ABP document into midwifery-led continuity of care, and iterative adaptation. Following the updated MRC framework, this study will employ a sequential mixed-methods design encompassing systematic review, participatory instrument development, Delphi consensus on the knowledge base, iterative technical development with usability testing, and a feasibility study across two perinatal centres in Bavaria. **Results/Conclusions**: FAIR-Birth is expected to generate a content-validated ABP instrument, a domain-restricted multilingual LLM dialogue system, and an evaluated application prototype. The work corresponds to the development and feasibility phases of the MRC framework; effectiveness questions are reserved for a planned subsequent randomised controlled trial.

## 1. Introduction

Clinical childbirth in high-income countries is increasingly shaped by standardised routines that do not always accommodate individual needs. In Germany, more than 690,000 births occurred in 2023; 98% took place in clinical settings, frequently attended by previously unknown obstetric staff [1,2]. Limmer and colleagues, in a sample purposively recruited for high exposure to disrespectful care, reported obstetric interventions performed without full informed consent in up to 43% of hospital births [3]. Approximately one in eight pregnant persons experiences clinically significant childbirth-related post-traumatic stress disorder (CB-PTSD) symptoms, with full CB-PTSD developing in between 4% and 12.5% of cases, depending on the measurement approach [4,5]. Associated effects include impaired mother-infant bonding, breastfeeding difficulties, and strained partnerships [4].

Evidence on preference integration in birth care points in a consistent direction: integrating the birthing person’s preferences into the birth process is associated with higher satisfaction, fewer unnecessary interventions, and improved birth experience [6,7,8]. Midwifery-led continuity-of-care models are associated with lower caesarean section rates and improved maternal outcomes [9].

Birth plans are an established but imperfect instrument for the purpose of preference articulation. A systematic integrative review found that while birth plans can increase discussion of preferences with care providers, they are frequently too static and too detailed to accommodate the dynamic nature of labour [10]. Existing digital tools focus predominantly on the care provider’s side of the encounter [11]. Several existing approaches address parts of this gap. The Birth Map is a structured, yet user-oriented and flexible, woman-led birth-preparation tool in book format, empirically evaluated in an Australian context, but it is English-only, static in format, and not integrated into the clinical record [12]. CHAT-maternity-care [11] addresses the care-provider side, supporting providers in assessing parents’ health literacy rather than supporting pregnant persons in articulating preferences. Conversational agents for perinatal health are an emerging field [13,14,15], with no instrument to date specifically structured around antenatal preference articulation. General-purpose LLMs are accessible but not domain-restricted; without grounding in validated content, their obstetric output risks being inaccurate or misread as clinical guidance [14]. FAIR-Birth is designed to bring these elements together—structured preference articulation on the pregnant-person side, digital adaptivity, multilingual access, and integration into midwifery-led continuity of care—within a single instrument.

Communication barriers compound this gap. Approximately 13.4% of the German population did not grow up speaking German as a first language [16]. Pregnant persons with migration history and limited German proficiency face elevated risks of inadequate informed consent and loss of autonomy during labour [17]. Midwifery students in Germany receive limited standardised training in the care of pregnant persons with language barriers, FGM/C (Female Genital Mutilation/Cutting), or experiences of racism—a curricular gap that may reinforce existing inequalities in clinical practice [18].

Digital health technologies offer an opportunity here. Conversational agents based on LLMs can deliver information and support shared decision-making at scale, adapting to a user’s language and health-literacy level [19]. What is missing is not digitalisation alone but a dynamic, adaptive instrument responsive to a pregnant person’s evolving preferences across successive encounters, which a domain-restricted LLM is suited to provide.

This paper presents the conceptual foundation and proposed study design for FAIR-Birth (Fair, AI-supported, Informed, Respectful), an interdisciplinary research initiative to develop and feasibility-test the FAIR-Birth intervention: a mobile application supporting Advance Birth Planning (ABP) embedded within midwifery-led antenatal care. The FAIR-Birth intervention combines four elements: the transfer of the Advance Care Planning (ACP) concept to the antenatal context; the use of a domain-restricted Large Language Model to support multilingual preference articulation; integration of the resulting ABP document into midwifery-led continuity of care; and iterative adaptation of preferences across antenatal encounters. Each component has precedents in the literature on ACP, on digital decision support in maternity care, and on midwifery-led care models. Because these four elements have previously only been evaluated in isolation, the primary contribution of FAIR-Birth lies in their operational integration within a unified, midwifery-led antenatal care workflow.

The acronym FAIR refers to the procedural equity principles underlying the project: that clinical birth should be transparent, participatory, and responsive to individual preferences regardless of linguistic or social background. Whether the FAIR-Birth intervention may produce equitable outcomes is a question for the planned trial.

This paper presents the protocol prior to formal funding and ethics approval. Both will be obtained before data collection commences. Publishing at this stage invites methodological scrutiny and establishes conceptual transparency before the intervention is built. The relationship of the study to the phases of the MRC framework for complex interventions is set out in Section 4.1.

The remainder of the paper is structured as follows: Section 2 develops the theoretical framework; Section 3 describes the FAIR-Birth intervention concept; Section 4 outlines the proposed methodology; and Section 5 discusses possible implications and limitations.

## 2. Theoretical Framework

### 2.1. From Advance Care Planning to Advance Birth Planning

Advance Care Planning (ACP) is a structured process through which individuals clarify, document, and communicate their values, goals, and preferences regarding future medical care, in dialogue with healthcare professionals and significant others [20]. Evidence indicates that ACP can improve concordance between patients’ expressed wishes and the care they receive, reduce decisional conflict, and increase satisfaction among patients, families, and care providers [21,22,23].

Transferring the ACP concept to childbirth is conceptually motivated and practically supported. Childbirth shares structural conditions with end-of-life care—high relevance for the individual, physiological intensity, time pressure, and an often-unfamiliar care team [3,10]—that constrain real-time deliberative decision-making. Documented preferences expressed in advance can be carried into encounters where the deliberation would otherwise occur under time pressure [24].

The analogy is, however, partial. ACP responds to anticipated cognitive or medical incapacity in life-limiting illness. ABP addresses the temporary erosion of deliberative space during a physiologically normal process. The pregnant person retains full legal decision-making capacity throughout; what is at risk is the practical conditions for its exercise under pain, urgency, and institutional power of the clinical team. ABP is therefore not a transfer of substituted-judgement logic but of the dialogic structure of ACP adapted to the antenatal setting.

A precedent procedure exists in perinatal palliative care, where Garten et al. extended ACP into a structured, prospective, revisable prenatal planning process for pregnancies affected by life-limiting foetal conditions [25]. FAIR-Birth draws on the same dialogic and documentary structure, but differs in two respects: it addresses physiologically normal birth, and it centres on the competent pregnant person’s own preferences rather than anticipatory decision-making by parents on behalf of a child.

ABP adapts the dialogic structure of ACP to the antenatal routine context. It focuses on values and preferences regarding birth, rather than incapacity scenarios. Its output is a structured articulation of preferences rather than a static birth plan. Preferences are revised iteratively across antenatal encounters. The process is integrated into midwifery-led continuity-of-care, the relational context ACP research identifies as central to the concept’s effects [23].

### 2.2. Shared Decision-Making in Midwifery-Led Intrapartum Care

Shared decision-making (SDM), the joint process by which patients and healthcare professionals participate in clinical decisions, has become a normative standard in healthcare policy [26,27,28]. The 2020 German S3 guideline on physiological vaginal birth at due date explicitly calls for integrating women’s perspectives into clinical decision-making [29]. In obstetrics, SDM faces practical barriers: decisions are often required rapidly, under physical and emotional intensity, within a care relationship that may have formed only moments before [30].

ABP relocates the deliberative phase of SDM to the antenatal period, where the pregnant person has the time and cognitive space for structured reflection. The counselling midwife acts as a facilitator rather than a decision-maker. The ABP document may then provide an SDM infrastructure that persists across encounters, refined at successive antenatal appointments, stored in the electronic patient record (TI-ePA), and retrieved by the clinical team at birth. The FAIR-Birth application thus functions as a preparation and communication infrastructure.

### 2.3. Health Equity and the FAIR Framework

The third theoretical pillar of FAIR-Birth is health equity, understood procedurally. Procedural equity concerns whether each pregnant person has genuine access to the informational and deliberative resources required to exercise autonomous choice—not whether outcomes are equal across groups. Drawing on Rawlsian fairness [31,32], fairness of a care encounter depends not on whether the same procedure was offered to everyone but whether each pregnant person could reflect on, articulate, and communicate their preferences.

This procedural framing has empirical correlates that a feasibility study can examine, even if outcome-level equity cannot yet be tested. Knowledge of healthcare rights and perception of available resources during pregnancy and birth are unequally distributed, particularly among pregnant persons with undesired pregnancies [33]. Those with limited health literacy are underserved by existing birth plan formats [10] and those with migration history face language and system unfamiliarity [17].

FAIR-Birth operationalises procedural equity through three design choices. Firstly, the ABP instrument and application are multilingual by design, supported by an LLM adapting to the user’s language and health literacy level [34,35]. Secondly, the information component follows standards for evidence-based health communication [36,37]. Thirdly, the participatory development process involves pregnant persons, midwives, and clinical partners as co-designers [38].

Whether these choices produce more equitable outcomes is a question for the planned trial. The feasibility study examines whether the equity-supporting infrastructure can be implemented and used.

A positive birth experience is defined less by the absence of intervention than by the degree to which a pregnant person feels heard, informed, and in control [6,39], the conditions FAIR-Birth is designed to support.

## 3. The FAIR-Birth Intervention Concept

### 3.1. The Advance Birth Planning Instrument

**Figure 1:**
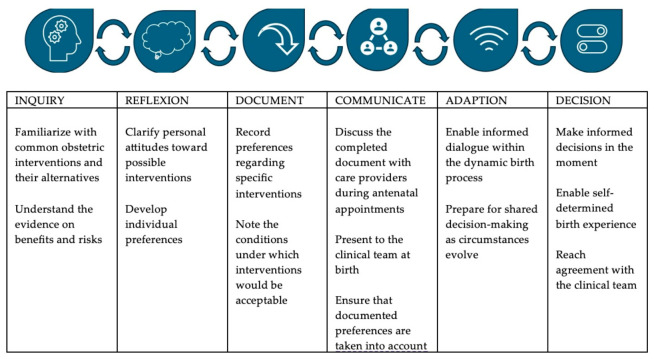
Simplified schematic of the Advance Birth Planning process.

The ABP instrument is the core preference-articulation component of the FAIR-Birth intervention. It is a structured, self-reflective dialogue guide that leads pregnant persons through a staged process of identifying, articulating, and documenting their preferences and goals for clinical birth. The process is organised into six domains, addressed sequentially and presented in Figure 1.

The six-domain architecture, adapted from [25], reflects the iterative and non-linear nature of antenatal preference formation. Birth preferences are not fixed at the outset of pregnancy; they emerge through dialogue, information, and self-reflection over time [40]. The ABP instrument is therefore a structured framework for an ongoing reflective process rather than a one-time exercise. It produces an ABP document recording current preferences and the conditions under which specific interventions would be acceptable, revised as preferences develop.

The ABP instrument complements existing care-provider-side tools by addressing the pregnant person’s side of the same encounter, equipping the pregnant person to articulate and communicate their own preferences. Together, the tools may support shared decision-making by giving both parties structured preparation for the antenatal dialogue within a midwifery-led care model.

### 3.2. The FAIR-Birth Application: LLM Architecture and Knowledge Infrastructure

The FAIR-Birth system will deliver the ABP instrument digitally and make evidence-based information about obstetric interventions accessible in a personalised, multilingual, and dialogically adaptive format.

**Figure 2:**
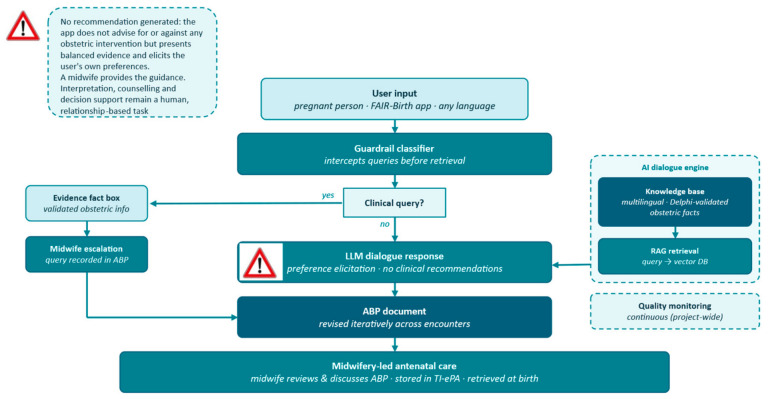
The FAIR-Birth system architecture.

Instrument development will follow an iterative participatory process. Focus groups with pregnant persons, practising midwives, and obstetric clinicians will generate design principles and content requirements. Cross-cultural validation across languages is planned to follow established methodological COSMIN standards [41,42]. The application’s information architecture will rest on a curated multilingual knowledge base covering common obstetric interventions (induction of labour, pain management, episiotomy, and caesarean section), as presented in Figure 2, developed through a systematic literature review and structured evidence synthesis and validated through a Delphi consensus process. Information follows established standards for evidence-based health communication, including evidence fact boxes [37] and the German guideline for evidence-based health information [36]. An LLM makes this knowledge base dynamically accessible. A core design choice is that the LLM does not generate clinical recommendations. Its function will merely be to facilitate the identification and structured articulation of individual preferences by presenting relevant information in the user’s preferred language at an appropriate health literacy level and guiding the reflective dialogue that produces the ABP document. FAIR-Birth is intended to serve as a preference elicitation and communication tool, not a clinical decision support system. This design choice shall address a core limitation of general-purpose LLMs in obstetric contexts. Without domain restriction, models such as ChatGPT 5.5 risk generating inaccurate, incomplete, or contextually inappropriate responses and may be misread by users as clinical guidance [14]. The FAIR-Birth system is designed to mitigate this through its curated knowledge base and Retrieval Augmented Generation (RAG) architecture, which may confine the model’s output to validated content. For direct clinical queries, the system employs a multi-layered risk-mitigation strategy. A guardrail classifier intercepts requests for clinical advice before retrieval; rather than generating a recommendation, the system redirects the query to the relevant evidence fact box, prompts reflection on the user’s own values, and records it in the ABP document for discussion with the midwife. Substantive clinical questions are thus routed to a qualified professional while the system continues to support structured preference articulation. Figure 2 gives an overview of the system architecture.

The LLM integration will follow a staged, non-fine-tuning adaptation strategy. Initial deployment shall employ RAG [43], in which the model queries a vector database of validated content prior to response generation, preventing output beyond the knowledge base. Task-specific behaviour is refined through advanced RAG techniques (query transformation, re-ranking), iterative system-prompt adaptation, few-shot prompting, and inference-time steering vectors [44,45]. In-context and test-time interventions require no participant interaction data for training. Reinforcement learning from human feedback and parameter-efficient fine-tuning are deferred to the subsequent randomised controlled trial, where a larger dataset and a dedicated consent process can be designed. The LLM component will be subject to continuous quality monitoring throughout the project period, comprising the instruction-following rate (accuracy), human-preferences alignment scores, and refusal rates under adversarial conditions. Full behavioural validation of the system across sites is a development-phase activity feeding the planned randomised controlled trial, rather than a confirmatory exercise completed within this protocol.

To provide an operational overview of the system’s technical boundaries, key implementation decisions, safety guardrails, and quality tracking metrics are structured in Table 1.

Transparency, explainability, and privacy are embedded in the system architecture from inception [46]. The FAIR-Birth application is being designed to meet the requirements applying to AI in healthcare through documented system behaviour, continuous performance monitoring, and expert-validated content.

### 3.3. Embedding FAIR-Birth in Midwifery-Led Antenatal Care

The FAIR-Birth intervention is not designed as a standalone person-facing technology. It is an ABP instrument and an ABP document, delivered through the FAIR-Birth application, embedded within midwifery-led antenatal care. The midwife’s contribution will be central to the intervention: the midwife has to introduce the ABP process at an antenatal appointment, facilitate discussion of the developing ABP document at subsequent appointments, and support its integration into the clinical record. This model is consistent with evidence on the effectiveness of midwife-led continuity of care [9] and with the broader move toward person-centred, relationship-based perinatal care [47].

Once completed and discussed, the ABP document is stored in the electronic patient record (TI-ePA) and available to the clinical team at birth. It gives the birthing person’s prior-articulated preferences a route into a care encounter where the team is otherwise meeting them for the first time [12].

## 4. Proposed Development and Evaluation Methodology

The proposed methodology will develop and feasibility-test the FAIR-Birth intervention in two phases. Phase 1 covers systematic development of the ABP instrument, the knowledge base supporting the FAIR-Birth application, and the technical platform itself, through participatory and iterative methods. Phase 2 examines whether the integrated FAIR-Birth intervention is feasible and acceptable in routine midwifery-led antenatal care, and generates the parameters needed to plan a future randomised controlled trial. The following section describes the designed methodological framework (Section 4.1), the planned four Phase 1 workstreams (Section 4.2), and the planned Phase 2 feasibility study (Section 4.3).

### 4.1. Methodological Framework

The FAIR-Birth intervention is conceptualised as a complex intervention in the sense of the updated Medical Research Council (MRC) framework [48]. Complex interventions have multiple interacting components, variable outcomes, and are sensitive to context and to the agency of those who implement them [49]. The FAIR-Birth intervention aims to meet these criteria: it comprises a digital tool, a structured dialogue process, a clinical workflow integration within midwifery-led care, and a multilingual knowledge infrastructure that together support pregnant persons in articulating birth preferences.

The study corresponds to Phase 1 (development) and Phase 2 (feasibility and piloting) of the MRC framework. Phase 3 (full RCT evaluation) is the intended follow-on research. Ethical analysis is integrated with empirical inquiry as concurrent rather than sequential activities, following Kuehlmeyer et al. [50]. Questions about the application’s legitimate scope, the consent procedures for participants with limited German or digital literacy, and the use of preference data for system refinement will be addressed within technical and clinical workstreams (Section 4.2 workstream 4, Section 4.3).

### 4.2. Phase 1: Systematic Development (Months 1–18)

- Workstream 1: Systematic literature review

A systematic review will establish the evidence base and identify design principles. The review will follow PRISMA 2020 guidelines [51] and address three questions: (a) What instruments exist for structured preference articulation in maternity care? (b) What design principles characterise effective instruments? (c) What evidence exists on their implementation feasibility? The review will include all study designs reporting on instruments or digital tools for birth-preference articulation. Methodological quality will be appraised with design-specific tools (CASP [52], MMAT [53], JBI [54]). Given the expected heterogeneity, no meta-analysis is planned; findings will be combined through structured narrative and thematic synthesis. Eligibility criteria, appraisal, and synthesis will be pre-specified in a dedicated review protocol, prospectively registered (e.g., PROSPERO), and reported per PRISMA-P.

- Workstream 2: Participatory instrument development

The guiding question is: What are the design principles and content requirements for the ABP instrument from the perspectives of key stakeholders? Three focus groups (n ≈ 6–10 each) will be conducted with purposively sampled stakeholders: pregnant persons aged 18 or older (varying in migration background and health literacy), practising midwives, and obstetric clinicians, recruited through antenatal clinics and cooperating midwifery practices. The focus groups are a participatory development activity generating design requirements; sample size is justified by information power [55] rather than by data saturation [56]. Analysis will follow a structured content-analytic approach, with reflexive thematic analysis [56,57] where deeper interpretive work is warranted. Patient and Public Involvement (PPI) will be integrated throughout [38]. A schematic logic model linking the intervention’s mechanisms, intermediate outputs, and feasibility outcomes will be developed collaboratively within this workstream and finalised prior to the Phase 2 feasibility study, ensuring it reflects the priorities of the participatory development partners rather than being specified a priori.

- Workstream 3: Knowledge base development and Delphi validation

A curated, multilingual knowledge base on common obstetric interventions will be developed through structured evidence synthesis, with expert validation to support clinical appropriateness across health literacy levels. A two-round online Delphi study (target n = 15–25 per round) will assess content validity and clinical appropriateness [58]. Consensus will be defined as ≥75% agreement per item. Items not reaching consensus in round one will be revised on panel feedback and re-rated in round two. The study team will recruit the panel from midwifery, obstetrics, and health-information specialisms; eligibility requires at least five years of relevant clinical or methodological experience, or a documented record in obstetric evidence synthesis or health-information development.

- Workstream 4: Technical development and usability testing

The LLM-based dialogue system will be developed iteratively from RAG-based deployment, as described in Section 3.2. This workstream addresses whether the FAIR-Birth application supports structured preference articulation in a way that is usable and acceptable for pregnant persons and midwives. Usability will be assessed with the MARS Scale [59] (target n = 10–15 per round, iterative refinement between rounds). The MARS was selected over general-purpose instruments such as the System Usability Scale because it was developed specifically for mobile health applications; scores will be interpreted descriptively against its subscale structure.

### 4.3. Phase 2: Feasibility Study (Months 19–30)

A prospective, mixed-methods feasibility study will be conducted with the developed ABP instrument across two perinatal centres in Bavaria. The study will follow a sequential explanatory mixed-methods design [60]. Quantitative feasibility data will be collected first and subsequently elaborated through qualitative inquiry.

- Setting and participants

Two level I/II perinatal centres in Upper Bavaria (approximately 1500–3000 births per year) have been identified as study sites. Eligible participants are pregnant persons in their second or third trimester in routine antenatal care; purposive sampling will ensure representation across migration history and health literacy. A target sample of n = 30–50 is planned. As this is a feasibility study, no power calculation is required; sample size is justified by the study’s objectives—estimating recruitment rates, assessing acceptability, and identifying procedural uncertainties for the planned trial [61].

- Quantitative feasibility outcomes

Quantitative feasibility data will be collected across four domains: recruitment (proportion of eligible and approached pregnant persons who consent; retention over the antenatal period), engagement with the FAIR-Birth application (completion of the ABP process; time spent in each ABP stage), procedural feasibility (data completeness; technical errors; midwife workload), and acceptability (Mobile App Rating Scale [MARS] [59] scores from pregnant persons and midwives). The four feasibility domains above (recruitment, engagement, procedural feasibility, and acceptability) constitute the primary feasibility signals informing the progression decision. The pilot psychometric measures introduced below are explicitly subordinate to a parameter-estimation role: they are collected to estimate variance and the plausible range of effect sizes for the future randomised controlled trial sample-size calculation, and are expressly not used to test effectiveness.

Equity-related process indicators will be collected alongside these outcomes: the proportion accessing application content in languages other than German, uptake of supported-access pathways (midwife-assisted onboarding, paper-based fallback), and the proportion recruited via midwifery practices serving underserved populations. Where cell sizes allow, completion and acceptability will be reported descriptively by language group, education, and migration history, without inferential comparison.

Pilot data on the Birth Satisfaction Scale-Revised (BSS-R) [62] and the Wijma Delivery Expectancy Questionnaire (W-DEQ_A) [63] will also be collected. These outcomes will not be used to test effectiveness in the feasibility phase. They will be used to estimate variance and the plausible range of effect sizes, informing the sample-size calculation for the planned randomised controlled trial.

- Progression decision

This study will not pre-specify numeric pass/fail thresholds. No prior empirical base exists for AI-supported antenatal preference articulation in multilingual midwifery-led care, so borrowed thresholds would be arbitrary; pre-specified thresholds also tend to penalise studies that sample underserved populations equitably [64]. The CONSORT extension for pilot and feasibility trials [65] frames such studies as informing future thresholds rather than passing or failing fixed ones.

The study will pre-specify a structured progression-decision process following Avery et al. [66]. At the end of Phase 2, the study team will review feasibility data, equity indicators, and qualitative interview findings against three possible verdicts: continue (recruitment achievable, application usable without structural redesign, ABP documentation adequate, midwife acceptability sufficient); amend (specific components require redesign before the RCT, but the intervention as a whole is feasible); or stop (a fundamental barrier cannot be resolved by amendment). The progression decision and the evidence supporting it will be documented in a progression report and published alongside the feasibility results, regardless of the verdict reached.

- Qualitative component

Semi-structured interviews with a purposive subsample of participants (target n = 12–15) and participating midwives (target n = 6–8) will explore experiences of the ABP process, perceived utility of the FAIR-Birth application in the midwifery encounter, and barriers and facilitators to implementation. The qualitative component is exploratory and illustrative; sample sizes are justified by information power [67] rather than by an expectation of thematic saturation. Data will be analysed using reflexive thematic analysis [57].

- Integration

Quantitative and qualitative data will be integrated at the interpretation stage using a joint display approach [68,69]. Qualitative findings will help to explain and contextualise the quantitative feasibility outcomes and generate recommendations for intervention refinement before the planned randomised controlled trial.

- Ethics and governance

Ethics approval will be sought from the relevant institutional ethics committee prior to any data collection, in accordance with the Declaration of Helsinki and current DFG guidelines [70]. Informed written consent will be obtained from all participants. For participants with limited digital literacy, consent will be obtained through a midwife-assisted procedure in which the study information is explained verbally and questions are addressed before written consent is given. Where a participant’s preferred language is one for which application content is available but consent materials have not yet been formally validated, consent will be supported by a professional interpreter or a qualified bilingual midwife, and participation in that language will begin only once the participant has confirmed understanding; the languages affected will be documented as part of the feasibility findings. All data will be pseudonymised and stored in compliance with GDPR. The study will be prospectively registered in DRKS or ClinicalTrials.gov before recruitment commences.

Within the feasibility study, the LLM is adapted exclusively through non-training methods (Section 3.2), so participant interaction data are not used to train or fine-tune the model. The governance principles guiding this stage are transparency, explainability, and data protection. Data are pseudonymised and processed on TH Rosenheim or trusted research-cluster infrastructure, with no transfer to commercial AI providers outside the EU, and participants are informed of data handling within informed consent. Reinforcement learning from human feedback and parameter-efficient fine-tuning, if pursued at the subsequent randomised controlled trial stage, would be accompanied by a dedicated, explicit consent process for the use of interaction data in model adaptation, designed with adequate lead time.

## 5. Discussion

FAIR-Birth addresses a documented gap: the absence of a multilingual, digitally supported instrument for structured advance birth planning embedded within midwifery-led care. Its conceptual contribution lies not in any single component but in the combination of the four elements set out above, each with precedents in the literature [9,11,12,25]. The contribution is the integration and its empirical implementation.

Three aspects warrant further comment. First, equity. By designing multilinguality and health-literacy adaptation into the application from inception, the project targets the procedural conditions that may produce unequal birth experiences; Phase 2 will assess whether this infrastructure is feasible to implement and is taken up by participants (Section 4.3), while outcome-level equity is a question for the planned trial.

Second, the role of the midwife. FAIR-Birth does not position the application as a substitute for the midwife–pregnant person relationship; it prepares the pregnant person for the midwifery encounter, and the midwife gives the ABP document its clinical meaning through dialogue. A multigroup analysis of 293 midwives and 215 physicians in Germany found midwives rated interprofessional collaboration and equitable communication lower than physicians, most markedly for equitable-communication items [71], indicating team asymmetries that extend to the birthing person’s encounter. The ABP document gives the birthing person a documented, prior-articulated voice in that encounter.

Third, the LLM component requires careful framing of its limitations. Large Language Models are susceptible to hallucination and can reflect training-data biases; algorithmic performance in controlled conditions often fails to transfer to real-world settings, and such biases can reproduce health inequities for underrepresented populations [72]. FAIR-Birth mitigates these risks through a curated, expert-validated knowledge base accessed via Retrieval Augmented Generation, restriction of the LLM to preference elicitation rather than clinical recommendation, and continuous quality monitoring.

FAIR-Birth aims to contribute to the emerging ecosystem of participatory tools in maternity care. Where CHAT-maternity-care [11] supports providers in estimating parents’ health literacy, FAIR-Birth equips pregnant persons to articulate their preferences. Emerging evidence indicates that preferences and access to birth-related information are among the factors shaping how birthing persons experience labour and birth [73].

The most closely related empirical work is The Birth Map [12], an Australian realist evaluation of a woman-led, book-based birth-preparation tool (n = 32). That study supports the feasibility hypothesis underlying FAIR-Birth: pregnant persons engage with structured preparation tools and report improved communication with care providers. FAIR-Birth extends this approach in four respects: it is digital and adaptive rather than book-format; it is multilingual, adapting to health-literacy level through a domain-restricted Large Language Model; it is integrated into midwifery-led continuity of care and the electronic patient record; and it is grounded in the Advance Care Planning concept, which The Birth Map is not.

Several limitations should be acknowledged. Firstly, the ABP process assumes antenatal preference formation translates into intrapartum experience and decision-making, which has not been validated in the specific form proposed here, though ACP research and evidence on birth-related mindset offer analogous support [23,40]. Secondly, even carefully prepared preferences may require revision under clinical necessity; the ABP instrument is framed as a foundation for dialogue, not a binding contract, and the iterative adaptation stage accommodates this. Thirdly, the application presupposes smartphone access and digital literacy, which risks reproducing the inequalities the project addresses [74,75]. Strategies to mitigate this (supported-access pathways, low-bandwidth versions, paper-based fallback) will be examined as process indicators in the feasibility phase, as described in Section 4.3.

A fourth limitation concerns scope. Phase 2 assesses whether the intervention is feasible and acceptable in routine antenatal care and estimates parameters for a future randomised controlled trial; it is not designed to assess clinical effectiveness, and feasibility findings should be read accordingly. Relatedly, full behavioural validation of the LLM component across sites is reserved for the planned randomised controlled trial; the feasibility study informs but does not confirm system behaviour at scale.

## 6. Conclusions

FAIR-Birth proposes a theoretically grounded, methodologically structured approach to a documented challenge in clinical obstetrics: supporting pregnant persons in articulating their preferences for clinical birth and communicating them to the care team. Its conceptual contribution rests on the combination of the four elements rather than any single component.

This paper has established the conceptual and methodological foundation for the FAIR-Birth intervention. The development and feasibility study will generate the evidence needed to assess whether the intervention is acceptable, feasible, and sufficiently promising to warrant a full-scale randomised controlled trial; the progression decision will be published regardless of the verdict. If feasibility findings support continuation, FAIR-Birth could contribute a scalable multilingual instrument to midwifery-led antenatal care. Whether it reduces preventable birth trauma or advances equity in birth experience are questions for the planned trial.

## Figures and Tables

**Table 1:** Summary of FAIR-Birth LLM architecture, safety, and evaluation metrics.

Component	Preliminary Technical Decisions	Safety & Guardrail Architecture	Expected Performance & Monitoring Metrics
Knowledge Access	Retrieval-Augmented Generation (RAG) using a local vector database.	System-prompt constraints; confinement to expert-validated facts.	Instruction-following rate (accuracy tracking).
Dialogue Steering	Non-fine-tuning adaptation; inference-time steering and few-shot prompting.	Guardrail classifier intercepts direct clinical queries.	Adversarial refusal rates (intercepting out-of-scope requests).
Human Alignment	Dynamic adaptation to user language and health-literacy level.	Redirection of clinical questions to evidence fact boxes and human midwives.	Human-preference alignment scores.

## Data Availability

No new data were created or analysed in this study. Data sharing is not applicable to this article.

## Abbreviations

The following abbreviations are used in this manuscript:

ABP	Advance Birth Planning
ACP	Advance Care Planning
CB-PTSD	Childbirth-Related Post-Traumatic Stress Disorder
FGM/C	Female Genital Mutilation/Cutting
LLM	Large Language Model
MRC	Medical Research Council
PPI	Patient and Public Involvement
RAG	Retrieval Augmented Generation
SDM	Shared Decision-Making
TI-ePA	Telematikinfrastruktur Electronic Patient Record
